# Supplementary material for: The interactome of intact mitochondria by cross-linking mass spectrometry provides evidence for coexisting respiratory supercomplexes
Source: Mol Cell Proteomics. 2017 Dec 8;17(2):216–32. doi: 10.1074/mcp.RA117.000470 (PMC5795388; doi:10.1074/mcp.RA117.000470)
Supplement: Supplemental Data [file supp_RA117.000470_133922_0_supp_23978_fzffwf.zip › spectra_annotation/mito_DR_spectra_annotation/228-1-6-1-15-1.pdf]

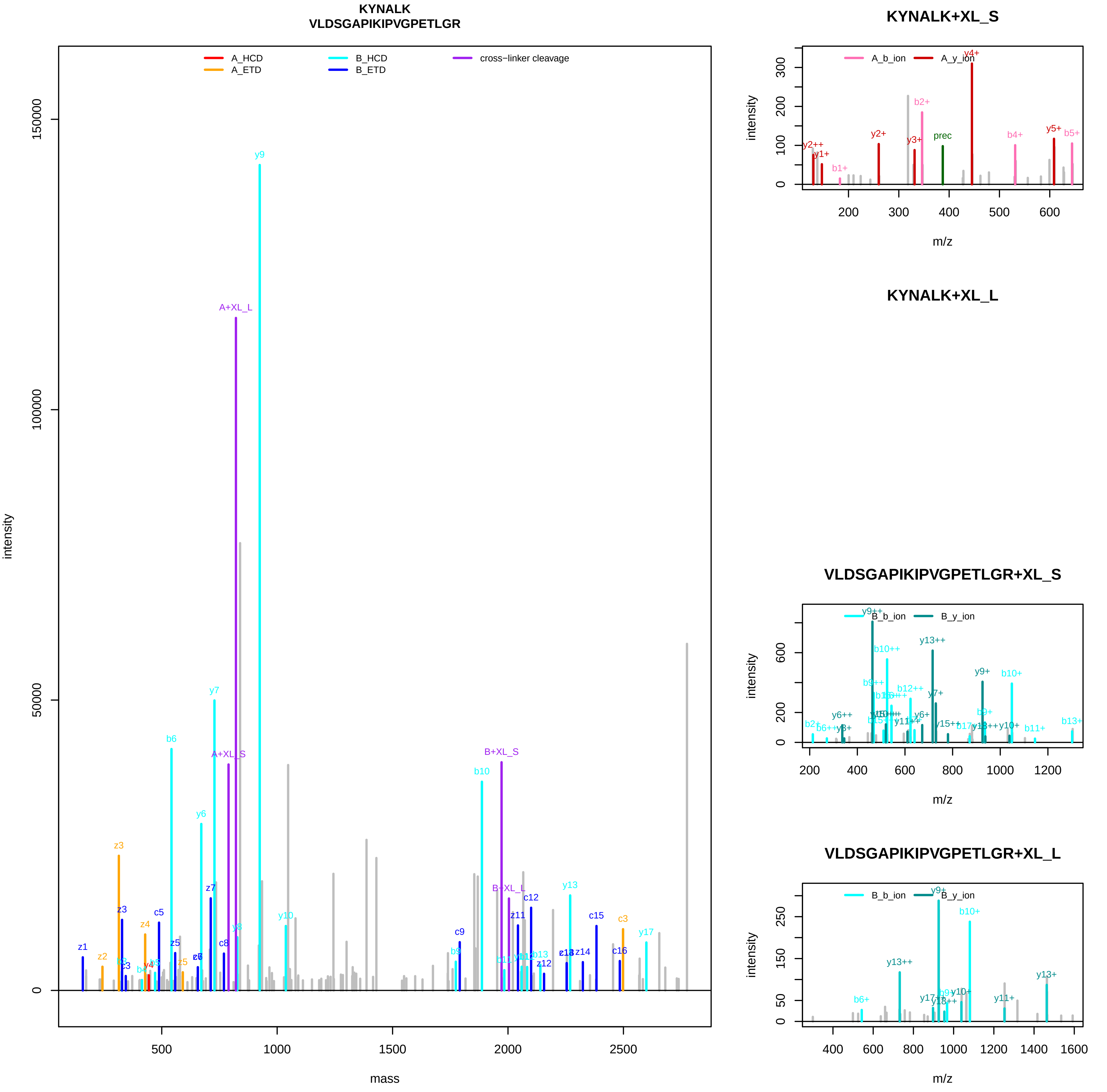

KYNALK
VLDSGAPIKIPVGPETLGR
A_HCD
A_ETD
B_HCD
B_ETD
cross−linker cleavage
intensity
mass
0
50000
100000
150000
500
1000
1500
2000
2500
y9
A+XL_L
y7
b6
A+XL_S
B+XL_S
b10
y6
z3
y13
B+XL_L
c12
z11
z7
c5
z4
y10
c15
c3
c9
y17
z1
z2
z5
c8
c16
z14
c13
z12
b13
KYNALK+XL_S
A_b_ion
A_y_ion
y4+
b2+
y2+
y3+
prec
b4+
y5+
b5+
y2++
y1+
b1+
m/z
KYNALK+XL_L
VLDSGAPIKIPVGPETLGR+XL_S
B_b_ion
B_y_ion
y9++
y13++
b10++
y9+
b10+
b9++
b12++
y7+
y6++
y6+
b2+
b13+
b11+
y10+
VLDSGAPIKIPVGPETLGR+XL_L
y13++
b6+
y11+
y13+
